# Probiotics and Phytobiotics as Dietary and Water Supplements in Biofloc Aquaculture Systems

**DOI:** 10.1155/anu/3089887

**Published:** 2024-12-11

**Authors:** Mohammad Hossein Khanjani, Moslem Sharifinia, Mohammad Akhavan-Bahabadi, Maurício Gustavo Coelho Emerenciano

**Affiliations:** ^1^Department of Fisheries Sciences and Engineering, Faculty of Natural Resources, University of Jiroft, Jiroft, Kerman, Iran; ^2^Shrimp Research Center, Iranian Fisheries Sciences Research Institute, Agricultural Research, Education and Extension Organization (AREEO), Bushehr 75169-89177, Iran; ^3^National Research Center of Saline-Waters Aquatics, Iranian Fisheries Science Research Institute (IFSRI), Agricultural Research, Education and Extension Organization (AREEO), Bafq, Yazd, Iran; ^4^Commonwealth Scientific and Industrial Research Organization (CSIRO), CSIRO Agriculture and Food, Livestock and Aquaculture Program, Aquaculture Systems Team, Bribie Island Research Centre, Woorim, Australia

**Keywords:** aquaculture, bioactive compounds, biofloc, phytobiotics, probiotics

## Abstract

Biofloc technology (BFT) is a relatively new microbial-based cultivation system that can be adopted to accomplish more sustainable aquaculture and circularity goals. This review explores aspects of BFT integrating the utilization of probiotics and phytobiotics as dietary and water supplements. This scientific-based snapshot unpacks some physiological pathways and brings a literature review on how these supplements can boost water quality, as well as aquatic species' growth, health, and survival. Probiotics, live microorganisms that confer health benefits on the host when administered in adequate dosage, are noted for their ability to bolster animal defenses and sustain water quality in farming conditions. Recent studies showcased that selected bacteria, yeast, and fungi, once added into biofloc-based systems can enhance animal performance, act as a tool for water quality management and protect fish and crustaceans against diseases. On the other hand, phytobiotics are additives sourced from plants that normally are added into compounded feeds and are known for their health and growth benefits in aquatic animals. These additives contain plant-based substances/extracts that play a key role to suppress inflammation, pathogens, and can also act as antioxidants. These selected ingredients can promote healthy gut microbiota, improve feed efficiency, and turn on genes responsible for immunity improving disease resistance of fish/shrimp. According to this review, the adoption of probiotics and phytobiotics in BFT can greatly increase farm outputs by producing healthier animals, as well as promoting growth and consistent yields. Lastly, this review showcases the importance of proper section of probiotics and phytobiotics in order to achieve a functioning BFT. Despite its numerous advantages, BFT faces several challenges, especially related to microbial management. Probiotics and phytobiotics are practical tools that can play a crucial role to obtain a more stable environment with a desirable microbial population in water and gut. Future directions in the field should focus on optimizing the utilization of these supplements for a more resilient and sustainable BFT aquaculture.

## 1. Introduction

Aquaculture is essential for supplying food and essential nutrients to a vast number of people globally, making it vital to tackle the global food shortage [1]. This could imply intensive aquaculture to cater to the needs of a swiftly expanding population, which comes with its own set of challenges [2, 3]. High stocking densities often lead to quick water quality deterioration, causing stress and increasing vulnerability to diseases, as these conditions create an ideal breeding ground for harmful pathogens [2, 4].

In traditional aquaculture systems, chemicals and drugs like antibiotics are commonly used to manage disease outbreaks in highly intensive fish and shrimp farming. However, this approach not only poses significant food safety risks due to harmful residues in the final product [4, 5], but it also contributes to the antibiotic-resistant bacteria-related issues in farmed fish and shrimp [6, 7]. To mitigate these issues, there is a growing need to reduce or eliminate the use of antibiotics and develop alternative management strategies to curb disease spread. Probiotics and phytobiotics are being investigated as potential alternatives [8, 9]. Additionally, commercial fish and shrimp farms are beginning to integrate biofloc technology (BFT) systems into their operations as a substitute for antibiotics [10–12].

BFT is increasingly being acknowledged for its environmental responsibility and sustainability, as it minimizes the requirement for water replacement and guarantees top-notch water purity in the intensive farming of fish and shellfish [13]. Numerous studies have reported the probiotic properties of bioflocs, which help exclude pathogenic microorganisms by creating a competitive environment and transforming otherwise toxic nitrogenous compounds into supplemental and nutritious food [14–16]. Furthermore, the simultaneous growth of heterotrophic bacteria and probiotics effectively inhibits the pathogenic bacteria growth [17, 18].

Utilizing biotechnologies like probiotics and bioflocs is a common in some countries practice to enhance output and lessen financial damages caused by viral and bacterial diseases [19]. Probiotics, live microorganisms that confer health and environmental benefits on the host when administered (water or feed) in suitable dosage, can be obtained commercially in either lyophilized or liquid forms [20]. Conversely, BFT encompasses a mix of algae, bacteria, fungi, detritus, and zooplankton as well, which are fostered by supplying organic carbon and nitrogen nutrients along with consistent water aeration [21]. These advancements offer the potential to alter the microbial ecosystem within the host, which can lead to better health in animals and an increase in water quality [22]. The enhancement in water quality and animal well-being enables a higher stocking density in nurseries, and bioflocs serve as a natural food supply [23]. Probiotics have been successfully integrated into the biofloc aquaculture system for various species of shrimp, such as giant freshwater prawn (*Macrobrachium rosenbergii*) [24], Pacific white shrimp (*Litopenaeus vannamei*) [25], and Indian white shrimp (*Penaeus indicus*) [26], as well as fish like Catfish (*Clarias gariepinus*) [27], Nile tilapia (*Oreochromis niloticus*) [28], and Snakehead fish (*Channa striata*) [29]. The combination of functional feed supplements and bioflocs has recently become a popular strategy in aquaculture operations [30, 31]. Studies have shown that BFT offers nutrient-rich food, enhances feed conversion efficiency, boosts productivity, and reduces aquaculture pollution [21]. Recent research has demonstrated that incorporating alternative items such as carbon sources or feed ingredients (e.g., mannooligosaccharides, pineapple, watermelon rind, orange peel-derived pectin, pizza by-products, and symbiotics) into the BFT greatly boosts the growth rate, immune system's ability to respond, and resistance to diseases in *O. niloticus* [32–37]. However, these tools must be assessed case by case as availability, biosecurity risks, and/or costs could become impeditive for their adoption.

Phytobiotics have been utilized in the BFT for shrimp species such as *M. rosenbergii* [38], *L. vannamei* [39], and various fish species including *O. niloticus* [30, 40, 41], and gourami (*Osphronemus gourami*) [12]. In addition, agricultural by-products have recently gained significant interest in aquaculture due to their potential as functional feed additives [42]. Over the past few decades, extensive research has confirmed that certain waste products (used to extract functional herbal compounds) can positively affect the growth, resistance, and immune systems of commercially farmed aquatic species when added in the feed [43, 44]. As the need for environmentally friendly and sustainable aquaculture practices grows, so does the interest in studying biofloc, probiotics, and phytobiotics in aquatic animals. While the individual use of biofloc, probiotics, and phytobiotics in aquaculture has become widespread, their combined use within a biofloc system has also seen growing recognition. Therefore, this review examines the role of probiotics and phytobiotics within the biofloc system, focusing on factors such as water quality, growth performance, survival rates, immune response, antioxidant activities, and pathogen resistance. It is important to mention that this review does not intend to provide any specific recommendation, for example, types of probiotics and strains that should be added in water, feed, or both; or in combination with phytobiotics. Instead, this scientific-based snapshot aims to unpack some physiological pathways and bring a literature review on how these supplements can impact the culture environment, as well as promote animal health and performance in different aquaculture scenarios and species.

## 2. BFT

The BFT is a significant advancement in aquaculture that leverages microbial biotechnology to enhance productivity and sustainability. It functions by converting toxic materials, especially nitrogen components, found in fish waste into useful products like protein, certain fatty acids (short-chain fatty acids) minerals, vitamins, and carotenoids which can then be used as supplementary feeds for fish and crustaceans [13, 45].

BFT systems are becoming increasingly popular among fish and shrimp farmers due to their ability to manage wastes and retain nutrients in the culture water [46]. They function by promoting the growth of beneficial bacterial communities within the culture water, which helps in breaking down organic carbon from the surrounding environment [47]. Bioflocs not only reduce the environmental footprint of production but also decrease feed costs [48]. However, the success of the biofloc system requires constant management (ensuring there is adequate aeration, daily additions of carbon and feed, measuring dissolved oxygen (DO), solids management, and ect). If not adequately controlled, this can make fish and shrimp more susceptible to diseases. Therefore, it is crucial for producers to understand and manage the microbial dynamics of the system. For example, it is important to maintain the concentration of suspended solids in the culture water within safe thresholds [21].

Despite the challenges, the benefits of BFT are numerous. The flocs are nutrient-rich in protein and a valuable source of phosphorus and vitamins [49]. The growth of these microbial flocs enhances water quality and traps harmful nitrogen, while biofloc production can reduce mortality, boost larval development, and elevate growth rates in the farmed animals [50]. Furthermore, the system's minimal water exchange requirement keeps the environmental footprint of production low [51].

## 3. Application of Probiotics in BFT

Numerous scientific investigations have been conducted with varying objectives to evaluate the efficacy of probiotics in fish and crustacean biofloc culture systems [14, 52, 53]. Several studies, as depicted in Table 1, have explored the incorporation of probiotics into fish/shrimp culture systems within biofloc.

### 3.1. Water Quality

The importance of effective water quality management and maintenance cannot be overstated for the successful cultivation of aquatic animals. Key water quality parameters such as pH, DO levels, oxidation–reduction potential (ORP), total suspended solids (TSSs), floc volume, nitrogen compounds, and the total *Vibrio* count (TVC) are vital for the BFT culture of aquatic animals [79].

The research by Amjad et al. [66] revealed that the addition of probiotics to the BFT culture system did not alter the pH of the culture water, but it led to a decrease in the levels of DO and ORP.

This may be due to the increased DO consumption by the culture organisms in the larger biofloc volumes present in the probiotic-treated groups. The DO levels in the BFT system culture water are primarily influenced by the consumption by culture organisms, aerobic heterotrophic microbes, and the microbial community of the biofloc [80, 81]. The authors suggest that the pH remained stable due to the addition of an excess of calcium carbonate (20 mg/L) to the tanks, which helps maintain high carbonate levels and pH buffering capacities throughout all the groups [66].

Earlier research produced comparable results: the use of probiotics did not change the pH compared to the control group that did not use probiotics [10]. Additionally, adding 20 ppm of calcium carbonate can maintain a pH above 8 in a marine biofloc system [82]. The study by Amjad et al. [66] indicates that the inclusion of probiotics resulted in larger floc volumes and, as a result, lower DO and ORP levels.

In the research by Hassan et al. [25], a system with minimal water exchange that utilized probiotics notably decreased total volatile compounds (TVCs), demonstrating a negative relationship. This discovery is consistent with the work of Kord et al. [83], who proposed that employing a variety of probiotic strains as water enhancements in *O. niloticus* farming could reduce TVC levels and help prevent fish disease. Additionally, Zhou et al. [84] discovered that *Bacillus* sp. probiotics in a water pond reduced the occurrence of *V. harveyi*, *V. vulnifcus*, *V. parahemolyticus*, and *V. vulnificus*. The most likely explanation is that probiotics actively absorb or decompose organic waste or toxins, thereby improving the environmental quality [85].

Numerous studies have shown that *Bacillus* probiotics can sustain DO levels within the ideal range [86, 87]. For instance, Hura et al. [86] found that *B. megaterium* enhanced DO levels during the transport of fish in their study on the cultivation of major carps in a clear water system. Similarly, Gomes et al. [87] and Zink et al. [88] observed that higher DO in *Bacillus*-supplemented waters during fish transport using various *Bacillus* species (*B. subtilis*, *B. licheniformis*, *B. megaterium*, and *B. laterosporous*). Nonetheless, no notable change in DO levels was noted when a mix of *B. megaterium* and *Streptomyces fradiae* was applied for water treatment [83]. It might be inferred that the studies on the control of DO by *Bacillus* probiotics are not as comprehensive as the research on their influence on nitrogenous compounds in aquaculture [89]. Additionally, probiotics like *Nitrifes*, Sulfur bacteria, *Bacillus* sp., *Pseudomonas* sp., *B. toyoi*, *S. fradiae* [90]; *Lactobacillus plantarum*, *L. casei* [91] could potentially increase DO concentration.

In the research by Hassan et al. [25], a system with minimal water exchange that included probiotics resulted in a notable reduction in water pH and, as a consequence, ammonia (NH_3_) levels. This was supported by a strong negative correlation between probiotics and both pH and NH_3_. The decrease in NH_3_ levels compared to the control group is in line with Kord et al. [83], who found a significant reduction in NH_3_ when various probiotics were added to Nile tilapia water ponds, attributing this to increased microbial activity that absorbed nitrogenous compounds and incorporated them into their metabolism. Additionally, the addition of probiotics to pond water may have enhanced the nitrifying bacteria population, aiding in the transformation of ammonia to nitrite and then to nitrate. This conclusion is consistent with Wang, Xu, and Xia [92], who found that introducing probiotics to shrimp ponds led to a substantial increase in the nitrifying bacteria *Nitrosomonas* and *Nitrobacter*. Studies conducted by Gomes et al. [87], Zink et al. [88], Khademzade et al. [93], and El-Kady et al. [7] have all demonstrated the efficacy of *Bacillus* species, including *B. subtilis*, *B. licheniformis*, *B. megaterium*, *B. laterosporous*, and *L. acidophilus*, in reducing nitrogenous compounds. These results are consistent with Cha et al. [94], who applied *B. subtilis* to reduce the ammonia nitrogen concentration in a Japanese flounder culture system. Introducing effective microbial communities into aquaculture ponds can improve the circulation of organic matter, maintaining a clean and healthy water environment for aquaculture. As per the study by Qiu et al. [24], the total nitrogen and ammonia nitrogen levels in water significantly reduced following the addition of *B. subtilis* and effective microorganisms (EMs), a finding that echoes the research of Cha et al. [94].

Research by Miao et al. [95] and Qiu et al. [24] showed that incorporating *B. subtilis* and beneficial microorganisms into biofloc aquaculture water enhanced the quality of *M. rosenbergii* aquaculture water. Similarly, Panigrahi et al. [26] noted a decrease in pH, TAN, NO_2_-N, NO_3_-N, and an increase in TDS and TSS in biofloc and biofloc supplemented with probiotics groups compared to the control group, which suggests an improvement in water quality. In the *P. vannamei* culture, the customized biofloc group (BFT-Probiotic, *B. tequilensis* AP) significantly reduced TAN compared to the control group. Additionally, a reduction in NO_3_ and DO was obtained in the probiotic-added BFT compared to the control [72].

Probiotics, specifically formulated for biofloc fish farming, are instrumental in enhancing the effectiveness of this technology. They aid in maintaining microbial balance, managing water quality, preventing diseases, and improving nutrient efficiency [19, 96, 97].

### 3.2. Growth Performance

The integration of probiotics within a biofloc culture has been shown to positively impact the growth of fish/shrimp [28, 66]. de Souza et al. [98] discovered that the growth performance of *Farfantepenaeus brasiliensis* could be boosted by incorporating probiotics (*Bacillus* sp. *Bacillus* sp., *Enterococcus* sp., *Lactobacillus* sp., *Bacillus cereus* var. *Toyoi*) into the BFT compared to the clear water group. Widanarni et al. [56] also reported similar results, showing that nursery culture supplemented with probiotics (probiotics bacteria mixture in concentration 10^8^ CFU/mL) resulted in markedly better growth performance in *L. vannamei*. From a production standpoint, it is clear that the improved growth performance and survival rate in the probiotic-treated group led to a significant increase in final biomass: a 70% increase in production (79 kg versus 46 kg) for the probiotic treatment compared to the control [14].

In BFT system, Qiu et al. [24] discovered that the growth performance and feeding efficiency of *M. rosenbergii* were markedly enhanced by the inclusion of *Bacillus subtilis* and EMs compared to those without bacterial additions. In BFT system, Amjad et al. [66] discovered that the inclusion of *B. amyloliquefaciens* and Yongstrong probiotics (which include *B. amyloliquefaciens* Ba-BPD1) significantly boosted the growth performance and feeding efficiency of *L. vannamei* compared to the control group.

Panigrahi et al. [26] recommended that probiotic bacteria (*Virgibacillus* sp., *Oceanobacillus* sp., *Bacillus* sp., *B. megaterium*, *B. marisflavi*, *Lysinibacillus*, *B. cereus*, *B. licheniformis*, and *B. subtilis*) supplementation in biofloc improved the growth performance and survival rate of *P. indicus*. Among the treatments, the supplementation of biofloc with a consortium of nine bacterial strains demonstrated superior survival and growth. Kim et al. [99] reported that the addition of *Bacillus* sp., *Lactobacillus* sp., and *Rhodobacter* sp. in biofloc could improve the growth of *Fennerpenaeus chinensis*, which result from the enhanced shrimp health by increasing immune responses and antioxidant abilities.

In BFT system, Amjad et al. [66] reported that the inclusion of *B. amyloliquefaciens* and Yongstrong probiotics (which include *B. amyloliquefaciens* Ba-BPD1) significantly enhanced the feed conversion ratio (FCR, 1.03) of *L. vannamei* compared to the control group (1.42). A lower FCR suggests greater absorption and growth efficiency at the same level of feed intake [100].

Nile tilapia grown in biofloc environments and treated with probiotic beneficial bacteria (*L. plantarum*, *B. subtilis*, *L. plantarum*, *L. rhamnosus*, *L. acidophilus*, *L. delbrueckii*) have demonstrated enhanced growth performance, feed efficiency and FCR (1.36–1.38) compared to the control group (1.55) [78].

This enhancement may be attributed to probiotics' rich nutrient content, including proteins and vitamins, which can support the nutritional needs of aquatic animals. Additionally, aquaculture water supplemented with probiotics modified the water environment's microbial flora, suppressed the growth and reproduction of harmful bacteria, and bolstered the immune system of shrimp/fish, thereby improving their immunity [99].

### 3.3. Immune and Antioxidant Indices

The integration of probiotics and biofloc in aquaculture has demonstrated its potential to enhance both innate immune and antioxidant activities [26, 58]. For instance, Miao et al. [101] employed *B. subtilis* and *Lactobacillus* sp. in the cultivation of *M. rosenbergii*, in conjunction with biofloc conditions, to enhance the immune response (by increasing the total hemocyte count, phagocytic activity, respiratory burst activity, serum activity of superoxide dismutase [SOD], and lysozyme) in compared to the control group. The incorporation of a commercial probiotic blend, comprising *Bacillus* sp., *Lactobacillus* sp., and *Rhodobacter* sp., into the BFT system bolstered the immune response, specifically prophenoloxidase (proPO), lysozyme, and serine proteinase, while mitigating oxidative stress in *F. chinensis* in compared to the control group [99].

Biofloc has the advantage of a food source all day available because it is more effective in animal growth than artificial feed as well as enhances animal health through the stimulation of immune system [21, 47].

There are a number of beneficial effects on the utilization of EMs in BFT in shrimp farming with controlling disease and environmental concerns [99, 101].

Analogous advantages were observed when *Bacillus* sp. was applied in *L. vannamei* culture within a superintensive system, resulting in an enhancement of immune status [102]. The direct use of *B. amyloliquefaciens* in biofloc water resulted in a boost to the shrimp's immune system by raising the proportion of granular hemocytes and the total hemolymph protein [103]. In the context of *F. brasiliensis* culture within a BFT, probiotics, particularly *B. cereus var. toyoi*, enhanced growth, survival, and biochemical composition [98]. Overall, *Bacillus* species have been substantiated as advantageous probiotic bacteria for enhancing immunity in cultured aquatic animal species [104, 105].

In a study by Panigrahi et al. [26], the nonspecific immune responses of *P. indicus*, including phenoloxidase activity, lysozyme activity, total hemocyte count, differential hemocyte counts, and phagocytic index, were assessed in response to biofloc and biofloc supplemented with nine *Bacillus* strains. The findings indicated that the bioaugmentation of specific *Bacillus* species under biofloc conditions enhances the shrimp's innate immune responses, such as the phenoloxidase enzyme, total and differential hemocyte count, phagocytosis, and lysozyme activity. Additionally, the lysozyme activity of serum in experimental shrimps was markedly increased by the addition of *B. cereus*, *B. licheniformis*, *B. subtilis* strain, and *Lysinibacillus*. Lysozyme is a nonspecific innate immune molecule that breaks down bacterial cells and prevents pathogens [106]. Similar conclusions were also reported by Vaseeharan and Ramasamy [107] in *P. monodon*, where the addition of *B. subtilis* strains increased lysozyme activity, providing protection against *V. harveyi* infection.

In a research conducted by Qiu et al. [24], the incorporation of *B. subtilis* into biofloc was found to enhance the plasma SOD levels and decrease the malonaldehyde (MDA) levels in *M. rosenbergii*. However, they found that the addition of beneficial microorganisms to the biofloc led to an increase in the MDA content in *M. rosenbergii* plasma, which may be due to the different effects of probiotics on dosage, the method of addition, and different feeding animals.

Additionally, the study revealed that the inclusion of *B. subtilis* and beneficial microorganisms in biofloc significantly boosted the plasma acid phosphatase and increased the plasma lysozyme levels in *M. rosenbergii*. Meanwhile, Panigrahi et al. [72] observed that the expression of genes associated with nonspecific immunity was upregulated in response to biofloc supplemented with *B. tequilensis* AP BFT3.

Panigrahi et al. [26] demonstrated that the supplementation of probiotic bacteria led to an increase in the expression of transglutaminase and proPO-activating enzyme genes in *P. indicus*. Moreover, Panigrahi et al. [72] discovered that Daxx (death domain-associated protein 6) in experimental shrimps (*L. vannamei*) was upregulated in response to the addition of biofloc and probiotics.

Daxx protein plays a role in innate immunity, silencing of this protein triggered mortality of *L. vannamei* up on challenged with *Vibrio parahaemolyticus* [72]. The observation of Daxx protein expression and an increased innate immune response in the studies indicates that the use of biofloc, along with the inclusion of probiotics, can effectively boost innate shrimp immunity. The results imply that biofloc, when combined with probiotics, generally strengthens the immune system in shrimps. This enhancement may be due to the extracellular metabolites (such as short-chain fatty acids, bacteriocins, and ect) produced by the probiotic bacteria. The cell surface antigens of probiotic bacteria, like peptidoglycan, can act as immunogens or may trigger immune responses in shrimp [108].

Bañuelos-Vargas et al. [77] verified that incorporating probiotics into the biofloc system led to a notable rise in the SOD and GPX enzyme activities in red tilapia. This elevated enzyme activity suggests the activation of the antioxidant defense within cells, offering protection against reactive oxygen species [109]. Mohammadi et al. [78] also conducted a study where Nile tilapia reared in biofloc units and treated with probiotics exhibited enhanced immune responses, antioxidant enzyme activities, and proinflammatory cytokine levels. It was noted that the respiratory activity was improved in tilapia, suggesting that the fish were immunized with phagocytic function in the leukocytes, enabling them to resist pathogens. Additionally, tilapia raised under biofloc and probiotic treatments exhibited increased myeloperoxidase (MPO), lysozyme, and ACH50 activities, which are linked to the antibacterial capabilities of the fish. The ACH50 activities were particularly high in groups treated with probiotics. Likewise, fish grown in biofloc units with the addition of exogenous probiotics showed enhanced respiratory, MPO, and lysozyme activities [110].

In summary, immunological research indicates that various probiotics can have distinct impacts on nonspecific immunity. This variation may be attributed to the different behaviors of probiotic bacteria under biofloc conditions. Beyond the cell wall components, these bacterial strains also exhibit differences in their enzyme production abilities and metabolic pathways [26]. Enzymatic probiotic strains are essential in the BFT, as they utilize the input carbohydrate and contribute to improved performance and immunity for the reared animals [58].

### 3.4. Disease Resistance

Table 2 presents the challenges posed by disease factors in the BFT during the adding of probiotics. Several researchers have suggested that the introduction of probiotic bacteria into biofloc culture systems is an effective approach for mitigating diseases in aquaculture [14, 66, 111, 115]. Studies have shown that *Bacillus* strains can enhance shrimp survival when exposed to pathogenic *Vibrio* in BFT [9] and clear water system [116].

Additionally, Van Doan et al. [112] showed that incorporating the probiotic *L. plantarum* into freshwater biofloc systems enhanced the growth of juvenile Nile tilapia and its resistance to *Streptococcus agalactiae*. Guo et al. [115] highlighted the critical role of native microbiota in safeguarding shrimp from bacterial pathogens and proposed a microecological regulation approach for creating probiotics to mitigate aquatic animal diseases. Azad et al. [117] administered the commercial probiotic Zymetin during the cultivation of juvenile prawns (*M. rosenbergii*) exposed to pathogenic strains of *Vibrio* sp. and *Aeromonas* sp., observing a notable increase in total and beneficial bacterial density and a significant decrease in certain harmful bacteria in the water and prawn intestine across all tested groups in clear water system. In a study by Vázquez-Euán et al. [111], it was determined that probiotics from various sources included in diets can modify the composition and structure of microbial communities in biofloc aquaculture systems, affecting both the water column and the gut microbiota of farmed *L. vannamei*. At the order level, the most abundant microorganisms in the BFT culture system were Rhodobacterales. The consumption of these diets led to an increase in the expression of genes BGBP and PO, associated with the shrimp's immune system, before and after exposure to WSSV and *V. parahaemolyticus*.

Amjad et al. [66] performed a bacterial challenge test by adding the pathogen *V. harveyi* to the culture water in a BFT with probiotics. The findings suggested that while some shrimp exhibited disease symptoms, the differences among all treatments were not significant, likely because of the high diversity and interactions of microbes in the biofloc, which aided in preventing infections and pathogen dissemination [118].

BFT system provides an excellent choice to add probiotics as natural feed, improving gut microbiota, increasing disease resistance, and growth of farmed aquatic animals [111].

## 4. Application of Phytobiotics in BFT

Prior research has shown that the association of biofloc with live microalgae can facilitate bioflocculation [119–121], enhance water quality [122], augment the nutritional content of the biofloc [119, 123], and boost the growth performance of the cultured organisms [124, 125].

Various studies have demonstrated the effectiveness of using phytobiotics in biofloc systems [31, 40, 126–128]. Some studies presented in Table 3 have explored the incorporation of phytobiotics into fish/shrimp culture systems within biofloc.

### 4.1. Water Quality

Microalgae are instrumental in water quality improvement, primarily through the degradation of ammonia, nitrate, and phosphates, as well as the production of oxygen via photosynthesis [142]. However, their inclusion in biofloc-based aquaculture systems does not seem to significantly affect dissolved inorganic nitrogen concentrations, implying that the benefits they offer may be modest and potentially influenced by microalgae densities. It also seems that microalgae might not be needed to remove ammonia because of the heterotrophs, this statement is supported by research of Pacheco-Vega et al. [59] and Fimbres-Acedo et al. [135].

In the context of BFT, studies on *L. vannamei* have shown that the adding of diatoms did not significantly alter TAN, NO_2_, and NO_3_ levels in the rearing media [59]. In contrast, research on biofloc-based Nile tilapia culture indicated that biofloc systems without microalgae had generally lower TAN and nitrate concentrations compared to those with *Chlorella* [135].

The alkalinity concentration in biofloc systems with microalgae addition was consistently higher than that of the control group, a trend also observed in biofloc-based shrimp culture when diatoms were present [59]. This suggests that microalgae may have lower alkalinity requirements compared to heterotrophic bacteria and nitrification bacteria, given that microalgae require ~0.88 mol alkalinity per mol ammonia, whereas heterotrophic bacteria require 1.0 mol alkalinity per mol ammonia, and nitrification bacteria require 1.97 mol alkalinity per mol ammonia [150].

The application of *Ulva lactuca* in biofloc systems has been reported to decrease TSS by 12.9% [151]. Intriguingly, the incorporation of bioflocs with seaweed resulted in a 26%–52% increase in settleable solids, potentially because of the colonization of microorganisms that use the seaweed as a substrate. Carvalho et al. [39] carried out a study where macroalgae, particularly *U. lactuca*, were grown in an integrated system with shrimp in a biofloc tank. A concentration of 1 g/L of *U. lactuca* showed effective nitrogen compound absorption (including NH_3_ and NO_3_), leading to reduced final concentrations of these nutrients at the end of cultivation compared to shrimp grown in monoculture conditions (without *U. Lactuca*). Moreover, Sarkar et al. [146] assessed an integrated method for raising *L. vannamei* with red seaweed *Agarophyton tenuistipitatum* in an experimental-scale biofloc system. NH_4_-N, NO_2_-N, NO_3_-N, and PO_4_-P were significantly reduced in biofloc-seaweed system compared to control and biofloc monoculture system. After 30 days of the experiment removal of NH_4_-N (93.73%), NO_2_-N (60.04%), NO_3_-N (73.38%), and PO_4_-P (49.06%) in biofloc-seaweed system was found significantly higher than biofloc monoculture system.

Jiménez-Ordaz et al. [132] proposed that diatoms contribute to the formation of biofloc by releasing mucilaginous substances that bind bacteria, forming biofloc, and providing nourishment for these microorganisms. These conclusions align with previous observations by Sanka, Suyono, and Alam [152] and Daglio et al. [153] on the involvement of diatoms in the process of biofloc formation.

Microalgae and bacteria can cohabit in aquatic ecosystems, often entering into mutually beneficial relationships. Microalgae provide oxygen, organic carbon, and other nutrients that foster bacterial growth. This cooperation is particularly noticeable in biofloc systems, particularly when *Chlorella* sp. is included, as indicated by the alterations in the physicochemical properties of biofloc particles and nutrient compositions. Various factors affect microbial aggregation in biofloc formation, such as the composition of microorganisms, the ionic composition of the medium, quorum sensing mechanisms among the microbes, and the presence of grazers [81]. Lee et al. [154] found that the inclusion of bacteria in a xenic culture of *Chlorella* sp. resulted in an increase in floc size, aiding in the sedimentation of *Chlorella* sp. biomass. Subsequent research showed that bacterial attachment to microalgae surfaces encourages extensive biofilm formation, which further boosts the attachment of microalgae cells to this layer, leading to larger floc sizes [121]. The addition of acclimated microalgae, such as *Nannochloris* sp., to biofloc systems can decrease the required carbon-to-nitrogen (C/N) ratio, thereby reducing the additional organic carbon needed for ammonia and nitrite removal, because the microalgae will utilize the ammonia and nitrate. This was shown in a study where *Nannochloris* sp. contributed to faster pollutant removal without nitrite accumulation, even at low C/N ratios [155]. Including acclimated microalgae like *Nannochloris* sp. in biofloc systems can lower the necessary C/N ratio, which in turn decreases the extra organic carbon required for the removal of ammonia and nitrate. This was demonstrated in a study where *Nannochloris* sp. facilitated quicker pollutant removal without nitrite build-up, even under low C/N ratios [155].

### 4.2. Growth Performance

The integration of microalgae and phytobiotic substances into biofloc aquaculture systems has been shown to improve the growth performance, survival, and FCR of farmed aquatic animals. Studies have suggested that microalgae, including *Chlorella* sp., can enhance the nutritional quality of bioflocs, which may boost the growth performance and feed utilization efficiency of freshwater prawns [38]. The presence of seaweed is recognized for its positive impact on shrimp growth, with research showing enhanced growth performance in tanks with seaweed as opposed to those without [113]. Similarly, incorporating diatoms into biofloc-based shrimp culture has been demonstrated to have a beneficial effect on growth performance [124, 125]. This implies that the advantages of adding microalgae may vary depending on the species of microalgae and the specific animals being cultivated. Biofloc-seaweed systems have been reported to increase the final weight of shrimp (*L. vannamei*) and survival percentages, as well as significantly increase the SGR of shrimp compared to biofloc systems alone [146].

The application of *C. vulgaris* as a feed supplement has been demonstrated to enhance the growth performance of *O. niloticus* in BFT tanks [141]. Additionally, it has been noted that the breakdown o*f C. vulgaris* in fish intestines stimulates the intestinal flora, leading to an increase in the activity of digestive enzymes and improved diet utilization, which contributes to a decrease in FCR [141]. However, as the fish biomass increased during the final growth stage, biofloc systems with microalgae additions saw reduced growth due to increased concentrations of NH_3_ and pH levels, suggesting that the effects of microalgae on the performance of biofloc systems may be affected by the nutrient load.

Recent studies have emphasized that incorporating a combination of microalgae species, including *Grammatophora* sp., *Navicula* sp., and *Schizochytrium* sp., into biofloc-based *L. vannamei* culture led to higher protein content in the biofloc and improved shrimp growth performance compared to using a single species of microalgae [132]. This indicates that the selection of microalgal species could be a crucial determinant of the microalgae's effectiveness in biofloc systems. De Araújo et al. [156] investigated the growth of *O. niloticus* in biofloc tanks with varying concentrations of *C. vulgaris* inoculation. Their findings indicated no significant differences in growth parameters after 63 days, suggesting that the species-specific effects of microalgae addition and also the density of microalgae may vary and should be carefully considered in the design of biofloc systems.

The study by Purbomartono, Hapsari, and Susanto [12] reveals that combining biofloc cultivation with the feed supplementation of ginger flour (*Zingiber officinale* Roscoe) can optimize the growth performance of gourami, with an effective dose of 5.63 g/kg feed. This suggests that the integration of ginger flour into biofloc systems can enhance the growth performance and feed efficiency of gourami effectively.

Lumsangkul et al. [127] found that feeding *O. niloticus* sugarcane bagasse (SB) in a BFT resulted in enhanced growth output and feed utilization. SB is recognized as a prebiotic source that can enhance fish growth and feed utilization when added in the feed, as supported by studies by Valladares-Diestra, de Souza Vandenberghe, and Soccol [157], Zhao et al. [158], and Sun et al. [159].

Van Doan et al. [30] reported that feeding *O. niloticus* varying amounts of coffee silverskin (CSS) led to substantial improvements in growth efficiency and FCR. Furthermore, Van Doan et al. [31] noted that Nile tilapia fed with 40 g/kg watermelon rind powder (WMRP) in a BFT significantly improved growth performance and FCR.

*O. niloticus* fed with 10 g/kg orange peels-derived pectin (OPDP) exhibited the best growth performance and the lowest FCR [35]. Similar positive effects were observed when *O. niloticus* were fed with 10 g/kg OPDP and *L. plantarum* (10^8^ CFU/g), resulting in improved growth performance and FCR in a indoor biofloc system for 12 weeks.

*O. niloticus* fed with 20 mg/kg Amla (*Phyllanthus emblica*) fruit extract (AFE) improved growth performance and FCR in a BFT for 8 weeks [37]. Wannavijit et al. [44] showed that supplementing longan seed powder (LS, 20 g/kg) in *O. niloticus* raised in a BFT led to higher growth performance and feed efficiency.

Herbal compounds can positively impact several fish species. Xuan et al. [128] reported that striped catfish (*Pangasianodon hypophthalmus*) fed with 40 g/kg rambutan (*Nephelium lappaceum* L.) peel powder (RP) in a BFT improved growth performance, feed efficiency, and survival rate for 8 weeks. *O. niloticus* fed with 10 g/kg rambutan seed (RS) improved growth performance, feed efficiency, and survival rate in a BFT for 8 weeks [145].

The observed improvement in growth parameters in fish fed diets supplemented with RS seems to be linked to the nutritional value and bioactive compounds present in the RS. RS contains 7.8%–14.10% protein by dry weight, which varies by variety [160]. It is also abundant in essential amino acids like lysine, leucine, valine, and isoleucine [161]. Furthermore, the seed is rich in polyunsaturated fatty acids, such as linoleic acid and eicosadienoic acid [162].

In general, the above studies showed that the addition of phytobiotics to the aquatic animal diet raised in the biofloc system improves growth performance, feed efficiency, and survival rate.

### 4.3. Immune and Antioxidant Indices

Microalgae, such as *C. vulgaris* and *Spirulina obliquus*, are capable of producing bioactive compounds with antioxidant and immunostimulant properties, including carotenoids and phenolic compounds. These compounds, such as *β*-carotene, lutein, vitamins E and C, have been shown to support the immune system in *O. niloticus* cultured in BFT [140].

Research by Lumsangkul et al. [127] found that *O. niloticus* fed with SB diets exhibited superior skin mucosal immunity compared to control. Supplementation of SB with fish diets was found to increase lysozyme and respiratory burst activities, likely due to the presence of flavonoids and phenolics in SB. These polyphenols have been shown to induce dendritic cells, modulate macrophage immunity, and enhance the proliferation of B and T cells [163]. The notable rise in the transcription of glutathione S-transferase (GST), glutathione peroxidase (GPX), and glutathione-disulfide reductase (GSR) in fish livers after SB supplementation indicates that the bioactive compounds in SB, such as xylooligosaccharides, which are potentially prebiotic, may contribute to the enhancement of the immune response of *O. niloticus* in biofloc tanks [164, 165].

In a study by Van Doan et al. [30], the incorporation of CSS into the diet of *O. niloticus* in a BFT led to an improved skin mucosal immune response and serum immune parameters. CSS contains bioactive compounds like galactomannans, arabinogalactans, and vitamin C, which have demonstrated immunostimulant properties and potential as prebiotics [166, 167].

Additionally, studies have shown that CSS contains polyphenols, melanoidins, and chlorogenic acids, all of which are known to enhance the immune system [168]. Other studies have also demonstrated the effectiveness of adding WMRP to the diets of *O. niloticus* in a BFT, leading to increased lysozyme and peroxidase activities in the skin mucus and improved serum immunity [31]. Furthermore, the supplementation of OPDP to the diets of *O. niloticus* in a biofloc system has been shown to significantly enhance the skin mucus lysozyme, peroxidase activities, serum lysozyme, and phagocytosis compared to controls [35]. The diet of 10 g/kg OPDP along with *L. plantarum* (108 CFU/g) was found to significantly stimulate the innate immune response of *O. niloticus* in a BFT [112].

The AFE supplementation in the diets of *O. niloticus* in a BFT has been shown to significantly enhance lysozyme activity in the serum and skin mucus samples, as well as improve phagocytic, alternative complement, respiratory burst activities, and peroxidase activity [37]. Similarly, LS supplementation in the diets of *O. niloticus* in a BFT system was found to improve immunological parameters in the serum and mucus of Nile tilapia [44].

The RS supplementation in the diets of *O. niloticus* raised in a BFT was found to improve immunological parameters (GST, GPX, and GSR) in the serum and mucus of *O. niloticus* [145].

### 4.4. Disease Resistance

The use of phytobiotics in biofloc systems has emerged as a promising strategy for controlling pathogens and diseases in aquaculture, with a focus on improving environmental conditions and the health of cultured animals [30, 31, 37]. Table 2 presents the challenges posed by disease factors in the BFT during the addition of phytobiotics. In a study by Van Doan et al. [30], it was found that *O. niloticus* exposed to *S. agalactiae* showed increased survival rates when fed with CSS. The highest survival rate of 83.33% was observed in groups fed 20 g/kg CSS, indicating that CSS may contribute to the control of microbial pathogens and infections, similar to the disease resistance provided by herbal diets [169, 170]. The enhancement in survival rates could be attributed to the improvement of immune parameters. Coffee by-products, including CSS, have been reported to contain antibacterial, antifungal, antioxidant, and anti-infectious components (including phenolic compounds, melanoidins, diterpenes, xanthines, and carotenoids) which are associated with therapeutic and pharmaceutical effects [171, 172].

Van Doan et al. [31] demonstrated the protective effects of WMRP against *S. agalactiae* in a biofloc system. Earlier work by Cemaluk [173] had shown that watermelon rind extracts could offer protection against various pathogenic bacteria, including *E. coli*, *Pseudomonas aeruginosa*, and *B. subtilis*. In another research by Van Doan et al. [35], OPDP was found to offer protection against *S. agalactiae* in a BFT, with different feeding levels of OPDP resulting in varying degrees of survival against the pathogen.

Continuous feeding with AFE for 8 weeks in a biofloc system allowed *O. niloticus* to resist *S. agalactiae* infection, as evidenced by a relative percent survival (RPS) ranging from 47.62% to 80.95%. Fish fed 20 mg/kg AFE showed the highest RPS and resistance to *S. agalactiae* [37]. These researches collectively suggest that the incorporation of phytobiotics into BFT can serve as an effective method for disease control in aquaculture. This approach could potentially enhance sustainability and reduce reliance on chemical treatments and medications.

## 5. Limitations of Using Probiotics and Phytobiotics

Effectiveness of probiotics can be compromised by other chemicals or drugs, potentially disrupting the establishment of beneficial microbes. Improper utilization and dosage of probiotics can impact the desirable microbial profile in water and gut, and in some circumstances be detrimental to the aquatic animals [174]. Additionally, the potential for lateral gene transfer of antibiotic resistance among commonly used probiotics, such as certain species of *Enterococcus*, *Lactobacillus*, and *Bacillus*, presents a challenge [175–177], antibiotic-resistant genes. In this sense, it is crucial to select probiotic strains properly and establish management guidelines, aiming to finetune types and dosage to provide suitable water quality (e.g., DO levels and pathogenic *Vibrio* populations) in the different BFT-based farming scenarios and species.

Phytobiotics, with their wide range of active ingredients, offer a promising alternative to antibiotics [178]. In the context of BFT (i.e., different farming conditions, species, microbial profiles, and water quality), the utilization of phytobiotics could provide variable results and impact their mechanisms of action. Other challenges may include potential side effects (e.g., presence of toxic or antinutritional compounds) and cost considerations. Effectiveness of some phytobiotics may vary based on factors such as herbal source, processing, active ingredients, and aquatic species. These variables may impact the quantification, standardization, and optimization of phytobiotics in aquafeeds, especially in complex microbial-based BFT culture conditions.

## 6. Conclusions

Different farming scenarios including farmed species, number of farms nearby, outdoor or indoor ponds/tanks, salinity, microbial profile fluctuations, stocking density, and occurrence of disease outbreaks are examples that will dictate the management of phytobiotics and probiotics. For this reason, this review did not intend to provide any specific recommendation (e.g., types of probiotics and strains added in water, feed or both; or in combination with different types of phytobiotics) acknowledging each farming scenario is different. Instead, this scientific-based snapshot unpacked some physiological mechanisms and brought a literature review on how these supplements can impact the culture environment, animal health, and performance.

Based on the studies presented, the integration of probiotics and phytobiotics into BFT systems may represent a significant advancement in terms of production consistency and resilience. These biotechnological tools may not only enhance the operational efficiency but also boost the eco-friendly approach in BFT-based aquaculture. The effective use of probiotics and phytobiotics in aquaculture requires careful consideration of factors such as species compatibility, population density, and accurate dosing regimens. To unlock the full potential of these biotechnological agents in BFT, more research is needed to better understand their ability to enrich the microbial community within the biofloc environment, as well as clarify the impact of herbal compounds in a complex microbial culture condition.

## Figures and Tables

**Table 1:** Addition of probiotics to biofloc aquaculture system.

Cultured species	IBW (g)	RP (day)	Probiotic species	Inclusion concentration	Main finding	Reference
*L. vannamei*	0.005	56	Probiotic A (*Bacillus*) Probiotic B (multi sp., *Bacillus* sp., *Pediococcus* sp., and *Enterococcus* sp.)	Probiotic A (5 × 10^10^ viable *Bacillus* spores/g); Probiotic B (multi sp. 2 × 10^9^ CFU/g)	↑Water quality. This improvement is achieved by reducing levels of TVC and ammonia (NH_3_), while simultaneously increasing the levels of DO	Hassan et al. [25]

*M. rosenbergii*	2.09	60	*B. subtilis*	10^9^ CFU/mL	↑ The health of the water body, the immune system, and the digestive process of *M. rosenbergii*. ↑The population of advantageous bacteria within the gut of *M. rosenbergii*	Qiu et al. [24]

*M. rosenbergii*	0.1	127	*Lactococcus lactis*	1 × 10^7^ CFU/mL	↑Growth rates and higher survival when subjected to biofloc and probiotic treatment	Cienfuegos-Martínez et al. [54]

*L. vannamei*	0.5	28	*L. plantarum*	2 × 10^8^ CFU/g	*P. vannamei* that were fed with *L. plantarum* supplemented diets did not display noticeable differences in their growth parameters compared to those on a control diet	Thompson et al. [55]

*L. vannamei*	15.39	25	Probiotics bacteria mixture	10^8^ CFU/mL every 5 days	↑ Shrimp growth and survival were particularly evident in the difference between the control group and the group treated with a C:N ratio of zero	Widanarni et al. [56]

*Penaeus indicus*	0.12	90	*Bacillus* group (*Virgibacillus* sp., *Oceanobacillus* sp., *Bacillus* sp., *B. megaterium*, *B. marisflavi*, *Lysinibacillus*, *B. cereus*, *B. licheniformis*, and *B. subtilis*	5.4 × 10^9^ CFU/mL	↑ The immunological parameters of shrimps, indicating a positive effect on their overall health and potentially making this method beneficial for improving cultivation conditions for *P. indicus*	Panigrahi et al. [26]

*L. vannamei*	0.0073	30–60	*Bacillus* sp., lactic acid, *Lactobacillus* sp., *Saccharomyces* sp.	80–120 ∈ 10^6^ CFU/mL	Within the biofloc system, native bacteria appear to have the most significant impact on bacterial diversity	Huerta-Rábago et al. [57]

*L. vannamei*	—	30	*B. subtilis*, *Bacillus licheniformis*, *Oceanobacillus*, and *Bacillus halosaccharomyces*	1 × 10^6^ CFU/mL	↑ A greater number of beneficial gut bacteria compared to controls. Some *Bacillus* strains identified were capable of producing all essential extracellular enzymes and demonstrated antibiotic film forming properties	Panigrahi et al. [58]

*L. vannamei*	—	70	Commercial probiotic-containing *Bacillus* sp.	1 × 10^9^ CFU/mL	↑ Water quality and the health of the shrimps. BFT treatment has been shown to promote the growth of beneficial bacteria that contribute to nutrient biosynthesis and degradation, the urea cycle, and fermentation processes, which can lead to improvements in water quality	Waiho et al. [11]

*L. vannamei*	0.42	44	*Schizochytrium* sp. and *Latobacillus plantarum*	Each tank was incubated with 800 L of microalgal culture and 100 L of probiotic bacterial culture at a density of 20 × 10^3^ cells/mL and 1 × 10^8^ CFU/mL	↑ Water quality and minimizes the need for periodic probiotic supplementation throughout the culture lifecycle	Pacheco-Vega et al. [59]

*L. vannamei*	3.29	27	*Lactobacillus casei*, *Bacillus* sp., *Pseudomonas* sp., *Nitrosomonas* sp., *Aerobacter* sp., *Nitrobacter* sp.	—	The research advocates for the incorporation of standalone probiotics into the culture of *L. vannamei* shrimp through a BFT. Evidence indicates that these probiotics effectively enhance the developmental progress of the shrimp	Kurniaji et al. [60]

*L. vannamei*	2	50	*L. plantarum*, *Bacillus* sp.	10^7^ CFU/mL of *L. plantarum*, 3.3 ∈ 10^7^ CFU/kg of *Bacillus* sp.	↓ The shrimp's survival rate, FCR, and water quality	Bolívar-Ramírez et al. [53]

*L. vannamei*	0.7	110	*B. infantis*	1 × 10^9^ CFU/g	*↑* Water quality and the zootechnical efficiency of *L. vannamei*	Okomoda et al. [61]

*L. vannamei*	0.002	61	*B. subtilis* and *B. licheniformis*	5 × 10^10^ CFU/g	*↑* Water quality, bacterial populations, phytoplankton, and growth performance of *L. vannamei*	Ferreira et al. [19]

*O. niloticus*	3.20	—	*Bacillus* sp.	Probiotic at a dose of 10 g per 100 m^2^ every 10 days	*↑* Growth performance, productivity, and sustainability of *O. niloticus* farming in earthen ponds	Phan et al. [62]

*C. gariepinus*	1.86	60	*Bacillus subtillis*, *Bacillus megaterium*, *Bacillus coagulans*, *B. cereus*, *Bacillus alvei*, *Bacillus amyloliquefaciens*, *Bacillus brevis*, *Bacillus circulans firmus*, *B. circulans*, *Bicillus pumilus*	2 g/m^3^	–The cultivation performance of fish. BFT that includes organic carbon appears to be more cost-effective and labor-efficient for fish farming compared to a system that also incorporates probiotics	Agusta, Zaidy, and Hasan [27]

*M. rosenbergii*	0.12	40	*B. subtilis* and *B. licheniformis*	1.08–3.25 × 10^5^ CFU/g	*↑* Survival rate of *M. rosenbergii*	Frozza et al. [63]

*L. vannamei*	10–12	90	Commercial probiotics: (PB1 and PB2, and one control PBN). The PB1 was composed by the mixture (50% : 50%) of Efinol PT (containing *Bacillus* sp., *lactic acid*, *Lactobacillus* sp., *Saccharomyces* sp.) and mix laboratory robles (containing a native microbial consortia)	80–120 × 10^6^ CFU/mL	–The water quality and the productive response of the shrimp	Arias-Moscoso et al. [10]

*L. vannamei*	6.4	75	*Exiguobacterium acetylicum*	1.58 ∈ 10^7^ CFU/mL	*↑* Survival rates (up to 90.9%) and productivity (increased to 1.42 kg/m³) among the shrimp	de Mello Júnior et al. [64]

*L. vannamei*	0.50	63	*B. subtilis*, *B. cereus*	4.00 × 10^8^ CFU/mL and 3.13 × 108 CFU/mL for strain NT9 and strain YB3, respectively	*B. cereus* YB3 facilitated the development of bioflocs, while *B. subtilis* NT9 contributed to enhancing the growth of shrimp	Huang et al. [65]

*L. vannamei*	0.06	40	*B. amyloliquefaciens*, P1, Yongstrong probiotics (containing *B. amyloliquefaciens* Ba-BPD1), and P2, BiomiXin probiotics (containing *B. amyloliquefaciens*)	1 × 10^9^ CFU/mL	*↑* Water quality, increase the size of the biofilm, and boost the bacterial count in the water, alongside improving the shrimp's growth	Amjad et al. [66]

*O. niloticus*	16.72	98	*B. subtilis* and *L. acidophilus*	1 × 10^7^ CFU/mL	The implementation of BFT systems has been shown to effectively augment TBC in both culture water and gut microbiota of the *O. niloticus*	Haraz et al. [67]

*L. vannamei*	0.19	42	*B. subtilis*	1 × 10^6^ CFU/mL	*↑* Water quality, intestinal digestive enzyme activity, and nonspecific immune enzymes activities of the shrimp	He et al. [68]

*O. niloticus*	2.2–2.7	210	*Lactobacillus* sp. *Lactobacillus rhamnosus* KU985435	2 × 10^10^ CFU/mL	*↑* Growth performance by optimizing feed absorption, improving blood chemistry, and enhancing overall health	Flefil et al. [28]

*L. vannamei*	0.39–0.84	56	*B. amyloliquefaciens*, *B. licheniformis*, *B. pumilus*, *B. subtilis*	3 × 10^9^ CFU/g	*↑* Survival rates, growth performance, and feed efficiency	Hussain et al. [69]

*L. vannamei*	38.47	—	Total heterotrophic bacterial counts	With sugarcane molasses treatment and wheat flour were estimated as 3.4 × 10^5^ CFU/mL and 1.2 × 105 CFU/mL, respectively	Wheat flour supplementation was superior for shrimp production and bacterial quality in the shrimp's food	Said and Ahmed [70]

Shrimp	—	—	B. *subtilis*, *B. licheniformis*, *B. pumilus*, *L. plantarum*, *L. acidophilus*, *Saccharomyces cerevisiae*, dextrose, yeast extract	—	*↑* The sustainability and food safety aspects of aquaculture. However, the effectiveness of these practices can be limited if complementary sustainable practices are not implemented alongside them	Vieira et al. [71]

*L. vannamei*	—	45	*Bacillus tequilensis*	10^9^ CFU/mL	*↑* The quality of the culture environment, production performances and ↓in the incidence of disease by stimulating the production of essential proteins	Panigrahi et al. [72]

*L. vannamei*	2	42	*B. amyloliquefaciens*	9.48 × 10^4^, 1.90 × 10^5^, and 3.79 × 10^5^	*↑* The immune system of shrimp by increasing the proportion of granular hemocytes and the total protein content in hemolymph. *↑* The immune performance against pathogens was enhanced due to the improved detection capabilities	Llario et al. [73]

*C. gariepinus*	12.31	49	Commercial probiotics (prod A, prod B, and prod C)	10 mL/m^3^	The use of Prod A at a dose of 10 mL/m^3^ on the media resulted in the highest productivity	Hartono and Barades [74]

*O. niloticus*	71.4	109	*B. velezensis*, *B. subitilis*	1–3.6 ∈ 10^7^ CFU/g	–The growth performance. Variations in results can occur depending on the probiotic concentration and administration method	Oliveira [75]
	5.34	90	*B. subtilis*	3.6–7.2 ∈ 10^4^ CFU/g		

*L. vannamei*	3.29	27	*L. casei*, *Bacillus* sp., *Pseudomonas* sp., *Nitrosomonas* sp., *Aerobacter* sp., *Nitrobacter* sp.	—	–The growth performance, feed conversion ratio, viability, floc volume, and water quality	Kurniaji et al. [60]

*C. striata*	—	60	*Lysinibacillus fusiformis*, *Bacillus* sp., and *B. subtilis*	*L. fusiformis* (1.9 × 10^7^), *Bacillus* sp., (3.8 × 10^6^), and *B. subtilis* (1.0 × 10^7^ CFU/mL)	The multispecies biofloc bacteria tested demonstrated outstanding performance in reactivation, multiplication, and application trials within a biofloc system. The bacterial density reached 10^8^ CFU/mL, indicating a robust bacterial population	Widyastuti et al. [29]

*Colossoma macropomum*	23.24	60	*B. subtilis*	4 × 10^8^ CFU/mL	*↑* The condition factor of the *O. niloticus*	Dos Santos et al. [76]

*O. niloticus*	71.4	112	*B. velezensis*, *B. subtilis*, *P. acidilactici*, *P. pentosaceus*, and *L. plantarum*	*B. velezensis* (1.39 × 10^10^ CFU/mL), *B. subtilis*, *P. acidilactici*, *P. pentosaceus*, and *L. plantarum* (3.6 × 10^4^ CFU/g)	*↑* Survival rates of *O. niloticus*	Padeniya et al. [9]

*Oreochromis* sp.	6.7	49	Commercial probiotic (BP1 and BP2), *B. subtilis*, *B. licheniformis*, *Lactobacillus brevis*, and *S. cerevisiae*	—	*↑* Immune and antioxidant responses, particularly at high stocking densities	Bañuelos-Vargas et al. [77]

*O. niloticus*	8.63	112	Single strain (*L. plantarum*) or multistrain (*B. subtilis*, *L. plantarum*, *L. rhamnosus*, *L. acidophilus*, *L. delbrueckii*)	10^8^ CFU/g	*↑* Growth rate, feed consumption, immune function, and activity of digestive and antioxidant enzymes	Mohammadi et al. [78]

*Note:* ↑, boosted; –, not affected; ↓, suppressed.

Abbreviations: CFU, colony-forming units; Do, dissolved oxygen; HDPE, high-density polyethylene, IW, initial weight; R-BFT, rapid-biofloc technology; RBFTM, rapid biofloc formation technology; RP, rearing period; TBC, total bacterial count; TVC, the total *Vibrio* count.

**Table 2:** Challenge with pathogens in the biofloc aquaculture system by adding probiotics and phytobiotics.

Cultured species	IBW (g)	Probiotic/phytobiotic	Challenge contidion	SR (%)	Main finding	Reference
*L. vannamei*	0.5	*L. plantarum*, *P. acidolactici*, and *L. curvatus* subsp. curvatus	*C. maltaromaticum*. Each shrimp was administered 1 × 10^7^ bacteria via intramuscular injection. Over 96 h	60%	↑ Suppression of harmful vibrios in BFT with probiotics	Thompson et al. [55]

*L. vannamei*	1.15	Multistrain probiotic product containing *Bacillus* sp., *Enterococcus* sp., *Thiobacillus* sp., and *Paracoccus* sp. with a total CFU of 2 × 10^9^/g, at a dosage of 0.5 ppm per week (final CFU 1 × 10^6^ L water) Multistrain probiotic product containing *Bacillus* sp., *Enterococcus* sp., and *Lactobacillus* sp. with a total CFU of 1 × 10^9^/g and was added daily to the feed at a dosage of 3 g/kg feed (total CFU 3 × 10^6^ g feed)	These nursed juveniles were all infected naturally by *V. parahaemolyticus* during the nursery phase	83% in probiotic—BFT, 52% in control group	↑ Controlling *V. parahaemolyticus* and ↑ growth rate and SR of shrimp in a BFT	Krummenauer et al. [14]

*L. vannamei*	17	—	Challenged with WSSV and *V. parahaemolyticus* by immersion in a solution with 1 ∈10^6^ UFC/mL	More than 80% in BFT treatments contain probiotics	↑ Resistance to pathogens and ↑SR of shrimp	Vázquez-Euán et al. [111]

*L. vannamei*	0.23	*B. amyloliquefaciens*, P1, and P2, BiomiXin probiotics (containing *B. amyloliquefaciens*)	10 mL of *V. harveyi* suspension (2 × 10^9^ CFU/mL) was added to each tank. Shrimp mortality recorded for 7 days after inoculation	80%–90%	–In the prevention of *V. harveyi* infections in *L. vannamei* shrimp	Amjad et al. [66]

*O. niloticus*	221–255	*B. velezensis* + *B. subtilis*, *P. acidilactici*, *P. pentosaceus*, and *L. plantarum*	At 14 weeks, the fish were challenged with a low dose of *Streptococcus iniae* (7.2 × 10^7^ CFU/mL, via intraperitoneal injection), infection for 6 days	93.7%–95%	↓ Mortality rates *O. niloticus*. In the expression of immune genes linked to diet during the initial period before the challenge and afterward when exposed to *S. iniae*	Padeniya et al. [9]
			At 16 weeks, the fish were challenged with a high dose of *S. iniae* (6.6 × 10^8^ CFU/ mL), infection for 10 days	65%–81.7%		

*O. niloticus*	8.63	Single strain (*L. plantarum*), multistrain (*B. subtilis*, *L. plantarum*, *L. rhamnosus*, *L. acidophilus*, *L. delbrueckii*)	Anesthetized fish (24 per treatment; 8 per tank) were intraperitoneally injected with 0.1 mL of fresh live *A. hydrophila* (10^8^ cells/mL), infection for 7 days	The highest fish SR was obtained in BFT with probiotics	↓ Mortality rates in *O. niloticus* in a BFT	Mohammadi et al. [78]

*O. niloticus*	15.54	Coffee silverskin (CSS)	Fish were intraperitoneally injected with 0.1 mL of 0.85% saline solution, consisting of 10^7^ CFU/mL of *S. agalactiae*, for 15 days	83.33%	CSS at a concentration of 20 g/kg provided the highest SR after testing against *S. agalactiae*	Van Doan et al. [30]

*O. niloticus*	17.14	Watermelon rind powder (WMRP)	Fish were intraperitoneally injected with 0.1 mL *S. agalactiae* (10^7^ CFU/mL of 0.85% saline solution), for 15 days	52.48%–76.2%	↑ SR in *O. niloticus* fed with 40 g/kg of WMRP after the challenge test against *S. agalactiae*	Van Doan et al. [31]

*O. niloticus*	9.09	Orange peels derived pectin (OPDP)	Fish were randomly selected and injected with 0.1 mL of 0.85% normal saline solution containing 10^7^ CFU/mL of *S. agalactiae*, for 15 days	45.45%–81.82%	↑ SR in *O. niloticus* fed with 10 g/kg of OPDP after the challenge test against *S. agalactiae*	Van Doan et al. [35]

*O. niloticus*	5.90	OPDP (0–10 g/kg) + *L. plantarum* (10^8^ CFU/g)	Fish were intraperitoneally injected with 0.1 mL *S. agalactiae* (10^7^ CFU/mL of 0.85% saline solution), for 15 days	43.33%–70%	↑ SR against *S. agalactiae*	Van Doan et al. [112]

*O. niloticus*	10.48	Diet supplemented with 0, 5, 10, 20, and 40 mg/kg Amla (*P. emblica*) fruit extract (AFE)	Fish were intraperitoneally injected with 0.1 mL *S. agalactiae* (10^7^ CFU/mL of 0.85% saline solution), for 15 days	47.62%–80.95%	↑ RPS and resistance to *S. agalactiae* in *O. niloticus* fed with 20 mg/kg of AFE	Van Doan et al. [37]

*O. niloticus*	12.77	Five experimental diets with inclusion levels of 0, 1, 2, 4, and 8 g/kg of chestnut (*Castanea sativa*) polyphenols (CSP)	Fish were intraperitoneally injected with 0.1 mL of 0.85% saline solution containing 10^7^ CFU/mL of *S. agalactiae*, for 15 days	37.5%–75%	↑ SR in *O. niloticus* that were fed diets enriched with 2 g (kg) of CSP	Van Doan et al. [36]

*L. vannamei*	2.63	*G. birdiae* and *G.domingensis*	WSSV	50%–56%	↑ Growth performance of *L. vannamei* and ↓ the levels of harmful *Cyanobacteria*	Brito et al. [113]

*L. vannamei*	—	*Neem* (*Azadirachta indica*)	20 L, neem extract (1 kg fresh neem left boiled in 30 L water)	—	↓ The population of detrimental *Vibrio* sp.	Mandal and Das [114]

*Note:* ↑, boosted; ↓, suppressed; –, not affected.

Abbreviations: IW, initial weight; RPS, relative percent survival; SR, survival rate.

**Table 3:** Addition of phytobiotics to biofloc aquaculture system.

Cultured species	IBW (g)	RP (day)	Phytobiotic	Inclusion concentration	Main finding	Reference
*M. rosenbergii*	0.25	60	*Chlorella* sp.	*Chlorella* sp. was added at the initiation of the experiment and every 2 weeks at 10% of the total volume to reach an estimated final density of about 10^5^/mL	↑ Growth performance in *M. rosenbergii* raised in a BFT	Ekasari et al. [38]

Shrimp	—	—	*Navicula* sp.	5 × 10^4^ cells/mL	The culture medium is made from residues of a shrimp culture biofloc, proving adequate for cultivating the microalgae species *Navicula* sp. This indicates that the presence of trace metals in the residue supports the growth of this algae	Abreu et al. [129]

*L. vannamei*	2.63	28	*Gracilaria birdiae* and *Gracilaria domingensis*	Seaweed stocked at a biomass of 2.0 kg/m^3^ (80 g of the fresh weight of seaweed per tank)	↑ The quality of the biofloc, ↓ the density of cyanobacteria and the FCR in *L. vannamei* reared in a BFT	Brito et al. [113]

	—	7	Six microalgal strains, no-bioflocculation (*Scenedesmus obliquus* and *Botryococcus braunii*), optimal bioflocculation (*Chlorella* sp. BWY-1, *Haematococcus pluvialis* and *Dictyosphaerium ehnenbergianum*), and overbioflocculation (*Chlorella vulgaris*)	Initial inoculation (0.08, 0.2, and 0.3 g/L)	*Chlorella* sp. BWY-1 is considered the optimal choice due to its exceptional ability to form effective bioflocs and promote the growth of organisms within them	Wang et al. [130]

*O. niloticus*	Nursery phase (0.33–60), grow-out phase (60–500)	21–40 weeks	*Chlorella* sp. *C. sorokiniana*	1.0 × 10^6^ cells/mL	↑ The performance of aquaculture but also facilitates the reuse of water and nutrients, thus creating a sustainable production system	Fimbres-Acedo et al. [131]

*L. vannamei*	1.4	42	Two diatoms: *Grammatophora* sp. and *Navicula* sp., and probiotic bacteria *Schizochytrium* sp. and *Lactobacillus fermentum* TD19	Microalgae were added at a concentration of 2 × 10^4^ cells/mL. The bacteria was added to each tank at an initial concentration of 3.5 × 10^3^ CFU/mL	↑ The formation of bioflocs and aid in the development of microbial communities that serve as live feed for shrimp	Jiménez-Ordaz et al. [132]

*L. vannamei* and *O. niloticus*	Shrimp (2.16), fish (1.53)	62	*S. obliquus*	Microalgae was added two times a week for a final concentration of 5 mg/L in the units	↑ Immunological state to the shrimp by boosting the total count of hemocytes and the concentration of serum proteins. ↑ Circulating monocytes and erythrocyte stability in the shrimp	Silva et al. [133]

	—	7	*Platymonas* sp.	Concentration 1 × 10^5^ cells/mL	↑ Water quality with a reduction in nitrogenous compounds and the creation of a suitable habitat for specific bacterial groups	Dong et al. [134]

*O. niloticus*	0.33	40	*Chlorella* sp., *Chlorella sorokiniana*, and *Chlorella sorokiniana*	Microalgae inoculation was performed on a weekly basis for each treatment at a concentration of 1.0 × 10^6^ cells/mL	↑ The initial nursery growth period and the subsequent expansion phase up to the 30th week in the culturing of *O. niloticus*	Fimbres-Acedo et al. [135]

*L. vannamei* and *O. niloticus*	Shrimp (2.16) and fish (1.53)	62	*S. obliquus*	The final *S. obliquus* concentration of 5 mg/L (dry biomass)	↑ Growth performance and productivity in *O. niloticus*	Silva et al. [136]

*L. vannamei*	—	—	*Arthrospira* (Spirulina) *platensis*	Microalgae additions to 1 × 10^5^ cell/mL every 4 days	↑ The rearing environment by ↓ nitrite nitrogen levels and ↑ the nursery performance for *L. vannamei*, particularly by ↑ the activities of antioxidant enzymes such as SOD, CAT, and peroxidase	Dong et al. [137]

Hybrid red tilapia (*O. mossambicus* ∈ *O. niloticus*)	14.5	50	*Oscillatoria* sp.	*Oscillatoria* sp. (0.0, 0.1, 0.5, and 1 mg/L)	The level of *Oscillatoria* sp. inoculum significantly influenced various parameters of a tilapia hybrid, including pH, chlorophyll a concentrations, TSS, NO_3_^−^ concentration, and overall productivity	Miranda-Baeza et al. [138]

*L. vannamei*	16.2	35	Diatoms (*Navicula* sp.) and rotifers (*Brachionus plicatilis*)	Diatoms were added on days 1, 5, 10, 15, 20, 25, and 30 at a density of 5 ∈ 10^4^ cells/mL (*Navicula* sp.) and 30 organisms/L (*B. plicatilis*)	↑ Superior performance metrics, underscoring their value as natural food options for the postlarvae of shrimp	Brito et al. [139]

*O. niloticus*	59.95	12	*C. vulgaris* and *S. obliquus*	Concentration of microalgae for inoculum was 0.014 g/L	↑ The health of *O. niloticus* by enhancing their immune response without negatively affecting survival, growth, or body composition when compared to control groups	Jung et al. [140]

*O. niloticus*	6.05–6.68	75	*C. vulgaris*	*C. vulgaris* (8 days incubation, 5 × 10^5^ cells/mL)	↑ Growth performance in *O. niloticus* in a BFT	Flefil, Aboseif, and Hussian [141]

*M. rosenbergii*	2	60	*Chlorella* sp. and *Ankistrodesmus* sp.	Microalgae addition was performed once a week with an final density in the rearing tank of about 10^5^ cell/mL	↑ In particle size and a modification of the fatty acid composition, but it did not have a detrimental effect on water quality and the growth of *M. rosenbergii*	Angela et al. [142]

	—	—	*Chlorella pyrenoidosa*	*C. pyrenoidosa* (0, 1 × 10^8^, 1 × 10^9^, 5 × 10^9^, and 1 × 10^10^ cells/L)	A *C. pyrenoidosa* concentration between 5 and 10 x 10^9^ cells/L to effectively and quickly develop bioflocs	Chen et al. [143]

*L. vannamei*	0.22	30	*Amphora coffeaeformis*, *Cylindrotheca closterium*, and *Conticribra weissflogii*	Cell density of 3 ∈ 10^4^ cells/L	↑ High cell density of diatoms in BFT systems. *A. coffeaeformis* and *C. weissflogii* diatoms might be more advantageous as food supplements for shrimp in biofloc nurseries than the pennate *C. closterium*	Martins et al. [119]

*L. vannamei*	0.016	20	*Chaetoceros calcitrans*, *Navicula* sp. and *Phaeodactylum tricornutum*	Diatoms were added on the 1st, 5th, 10th, and 15th day at a density of 5 ∈ 10^4^ cells/mL for each species	↑ Better growth in *L. vannamei* and aiding in the regulation of potentially damaging cyanobacteria populations	Marinho et al. [125]

*O. niloticus*	50.47	84	Broken rice flour (BRF) or broken wheat grain flour (BWGF)	—	↑ The growth and overall well-being of *O. niloticus* grown within a BFT	Zaki et al. [144]

*Osphronemus gourami*	—	90	Ginger (*Z. officinale* Roscoe) flour	Adding ginger flour in feed at a dose of 5.63 g/kg, 3.75 g/kg,1.88 g/kg feed, and control (T0)	↑ Growth performance of *O. gourami*, with an effective dose of 5.63 g/kg feed in a BFT	Purbomartono et al. [12]

*O. niloticus*	15.12	56	Sugarcane bagasse (SB)	Fish were provided a basal diet incorporated with SB at 10, 20, 40, and 80 g/kg	↑ Growth performance of *O. niloticus* at levels of 20 and 40 g/kg when compared to fish fed with baseline, 10, and 80 g/kg diets	Lumsangkul et al. [127]

*O. niloticus*	15.54	56	Coffee silverskin (CSS)	Adding CSS in feed at a dose of 10, 20, 40, and 80 g/kg feed, and control (without CSS)	↑ Innate immune responses of *O. niloticus* at level 20 g/kg of CSS in a BFT	Van Doan et al. [30]

*O. niloticus*	17.14	56	Watermelon rind powder (WMRP)	Adding WMRP in feed at a dose of 20, 40, 80, 160 g/kg feed, and control (without WMRP)	↑ Growth performance and health indicators of *O. niloticus* at level 40 g/kg of WMRP in a BFT	Van Doan et al. [31]

*O. niloticus*	9.09	56	Orange peels derived pectin (OPDP)	Fish were fed experimental diets containing different levels OPDP as follows: 0 (control in CW), 0 (control in BFT), 5, 10, and 20 g/kg OPDP	↑ Health status (mucus lysozyme and peroxidase activity) of *O. niloticus* at level 10 g/kg of OPDP in a BFT	Van Doan et al. [35]

*O. niloticus*	5.90	82	OPDP and *L. plantarum* CR1T5 (LP)	Fish were fed the following diets: diet 1 (0 g/kg OPDP and 0 CFU/g *L. plantarum*), diet 2 (10 g/kg OPDP), diet 3 (10^8^ CFU/g *L. plantarum*), and diet 4 (10 g/kg OPDP + 10^8^ CFU/g *L. plantarum*)	↑ Growth performance, innate immune responses, and offers protection against infections caused by *Streptococcus*	Van Doan et al. [112]

*O. niloticus*	10.48	56	Amla (*P. emblica*) fruit extract (AFE)	Fish were fed basal diet supplemented with 0, 5, 10, 20, and 40 mg/kg AFE	↑ Growth performance and innate immune responses in *O. niloticus* at level 20 mg/kg of AFE in a BFT	Van Doan et al. [37]

*O. niloticus*	12.77	56	Chestnut (*C. sativa*) polyphenols (CSP)	Five experimental diets with inclusion levels of 0, 1, 2, 4, and 8 g/kg of CSP were fed to Nile tilapia fingerlings	↑ Growth performance, innate immune responses, and disease resistance in *O. niloticus* at level 2 g/kg of CSP in a BFT	Van Doan et al. [36]

*O. niloticus*	13.82	56	Longan seed powder (LS)	Five diets, including the basal diet (control without LS) and basal diet containing 10, 20, 40, and 80 g/kg	↑ Growth performance, skin mucus, serum immune responses, and elevated expression of immune-related and antioxidant genes of *O. niloticus* at level 20 g/kg of LS in a BFT	Wannavijit et al. [44]

*P. hypophthalmus*	17.14	56	Rambutan (*N. lappaceum* L.) peel powder (RP)	Five diets: 0 g/kg (control) 10, 20, 40, and 80 g/kg	↑ Growth performance, skin mucus production, serum immunities, and upregulate immune-related gene expressions in *P. hypophthalmus* at level 40 g/kg of RP in a BFT	Xuan et al. [128]

*O. niloticus*	14.77	56	Rambutan (*N. lappaceum* L.) seed (RS)	Nile tilapia fingerlings were fed five experimental diets containing 0, 5, 10, 20, and 40 g/kg of RS	↑ Growth performance, skin mucus production, serum immunities, and the expression of genes related to immune defense against oxidative stress in *O. niloticus* at level 10 g/kg of RS in a BFT	Xuan et al. [145]

*L. vannamei*	6.2	35	*U. lactuca*	The densities of 1, 2, and 3 g/L of *U. lactuca* were cultivated in an integrated system with shrimp and monoculture treatment	Macroalgae can be successfully grown in a BFT system to facilitate nitrate uptake in a system that includes shrimp. When grown at a concentration of 1 g/L, macroalgae demonstrate improved nutrient absorption capabilities	Carvalho et al. [39]

*L. vannamei*	—	30	Red seaweed *Agarophyton tenuistipitatum*	—	↑ Water quality and growth performance of *L. vannamei*	Sarkar et al. [146]

*L. vannamei*	1.6	57	*Sarcocornia ambigua*	Evaluate the relationship between salinity in the performance of *L. vannamei* and *S. ambigua* in an aquaponic system with biofloc. Four treatments were evaluated: 8, 16, 24, and 32 psu	–In growth performance of *L. vannamei* within a salinity range of 16–24 psu. The *S. ambigua*, aiding in the removal of P and N compounds	Pinheiro et al. [147]

*O. niloticus*	3.15	70	*C. vulgaris*	*C. vulgaris* every 5 days at the concentration of 5 ∈ 10^4^ cells/mL	↑ Growth performance, feed efficiency, protein efficiency ratio, survival rate, and hematological indices of *O. niloticus* fingerlings cultured in low-salinity BFT	de Lima et al. [148]

*O. niloticus*	15.14	14	Black rice (*Oryza sativa L*.) bran (AR)	Five experimental AR diets (0, 1, 2, 4, and 8 g/kg)	↑ Growth performance, feed efficiency, mRNA transcript levels of innate immune, and antioxidant genes in *O. niloticus* grown in a BFT at concentrations of 4–8 g/kg AR	Linh et al. [149]

*O. niloticus*	14.15–14.25	56	Passionfruit (*Passiflora edulis*) peel powder (PSPP)	Fish were fed basal diets supplemented with different doses of PSPP at 10, 20, 40, and 80 g/kg	↑ The expression of innate immune and antioxidant-related genes in *O. niloticus* raised in a BFT at concentrations of 10–20 g/kg PSPP	Outama et al. [40]

*O. niloticus*	14.78	56	Mango peel powder (MGPP)	Fish were fed basal diet containing different levels MGPP as follows: 0, 6.25, 12.5, 25, and 50 g/kg diet	↑ Immune response and the expression of immune-related genes in *O. niloticus* at a dosage of 25 g/kg MGPP	Outama et al. [41]

*Note:* ↑, boosted; –, not affected; ↓, suppressed.

Abbreviations: ABFT, autotrophic biofloc technology; BFT, biofloc technology; CFU, colony-forming units; CW, clear water; DAPS, decoupled aquaponic systems; PSU, practical salinity unity; RP, rearing period; ST, sedimentation time; WC, water consumption; WSSV, white spot syndrome virus.

## Data Availability

No data were used for the research described in the article.
